# Professional Status of Audiology in U.S. Healthcare: A Health Economics Perspective

**DOI:** 10.1055/s-0046-1825794

**Published:** 2026-10-06

**Authors:** Amyn M. Amlani

**Affiliations:** Department of Communication Sciences and Disorders, College of Allied Health, University of Oklahoma Health Campus, Oklahoma City, Oklahoma, United States

**Keywords:** health economics, professional status, audiology workforce, reimbursement authority, healthcare labor markets

## Abstract

Professional status in healthcare is determined not only by educational preparation or statutory scope of practice, but also by how a profession functions within reimbursement systems, labor markets, and healthcare delivery. This article applies a health economics framework to evaluate the current professional status of audiology in the United States using observable economic indicators rather than aspirational goals or regulatory intent. Drawing on evidence synthesized from companion analyses of workforce trends, professional earnings, educational investment, reimbursement policy, and labor market dynamics, the review examines how reimbursement authority, pricing power, workforce growth, earnings trajectories, and productivity collectively reflect professional standing. The analysis demonstrates that, despite doctoral-level education and expanding clinical competencies, audiology remains constrained by limited reimbursement authority, restricted pricing autonomy, narrow service valuation, and clinician-dependent delivery models that limit workforce scalability and economic returns. These structural conditions contribute to constrained earnings growth, workforce instability, and increasing debt-to-income pressures, placing the profession in an intermediate economic position relative to other doctoral health professions. The article concludes that professional advancement should be evaluated through measurable economic outcomes and that meaningful progress toward autonomous professional status will require coordinated alignment of reimbursement, revenue architecture, labor deployment, and professional classification within the U.S. healthcare system.

## Introduction

Professional status in healthcare is, ultimately, determined not by stated intent, credentials, or scope-of-practice language, but by how a profession functions within payment systems, labor markets, and care delivery structures. Professional status matters because it shapes how care is accessed and sustained, the degree of authority to evaluate, diagnose, treat, and manage patients, how health systems deploy clinical labor, how payers value services, and whether a profession can support a workforce capable of meeting population health needs.

The role of health economics is to evaluate professional standing through revealed preferences rather than stated intent. As an example, reimbursement authority signals whether a profession is recognized as an independent provider or remains structurally dependent on intermediaries. Pricing power reflects the ability to value and bill services in proportion to training and clinical responsibility. Labor market outcomes—such as earnings growth, retention, and workforce expansion—indicate whether returns to human capital investment are sufficient to sustain long-term participation. When these economic signals align, professions tend to exhibit stable growth, scalable productivity, and durable integration across care pathways. When they do not, workforce strain, earnings compression, and professional ambiguity emerge—even in the presence of high levels of clinical expertise.

Audiology provides a particularly instructive case study. Over the past two decades, the profession has transitioned to doctoral-level entry, expanded clinical scope, and articulated a future vision centered on autonomy, integration, and workforce sustainability. From a training and competency standpoint, audiology increasingly resembles other doctoral-level health professions. Yet, many of the economic indicators typically associated with such professions—robust earnings growth, independent reimbursement authority, and scalable care models—have not materialized to the same degree. The result is a persistent divergence between educational investment and observed economic return.

Where companion analyses in this issue characterize the institutional architecture underlying these patterns (see Audiology at the Intersection of Labor, Revenue, and Professional Taxonomy), this manuscript applies a health economics framework to evaluate what that architecture reveals about audiology's present professional standing. By examining the alignment between training, economic authority, and labor market outcomes through observable economic signals rather than stated intent, this analysis offers a present-state diagnosis of audiology's position within the U.S. healthcare labor market and clarifies why forward-looking initiatives such as Audiology 2050 1 remain necessary. This analysis is intentionally diagnostic rather than prescriptive: the framework is used to assess where misalignments exist and what economic conditions would need to shift for professional autonomy to be realized in practice—not to advocate for specific reforms, evaluate the likelihood of legislative outcomes, or prescribe implementation sequences. That translational work is the function of the companion qualitative synthesis in this issue (see Audiology in 2050: Aligning Professional Prospectives to Create a Roadmap for the Future). The contribution of this manuscript is the economic diagnosis itself, applied through a replicable framework to observed market behavior rather than aspirational goals.

## Health Economics Framework for Assessing Professional Status

To assess audiology's current professional position, this article applies a structured health economics framework that evaluates how professional training, authority, and labor deployment interact within healthcare markets. Rather than restating normative definitions of professional status, the framework operationalizes status through observable economic relationships, organized around five interdependent dimensions that together determine whether a profession functions as an autonomous, sustainable provider of healthcare services: (1) human capital investment and economic return, (2) pricing and reimbursement authority, (3) market power and substitutability, (4) productivity, leverage, and scalability, and (5) earnings and wage growth as a composite signal. Each dimension captures a distinct mechanism through which professional authority is either reinforced or constrained—and together they form the analytic lens through which audiology's present position is diagnosed.

Comparative analysis is central to this framework. Examining audiology alongside other doctoral-level health professions provides a reference point for interpreting economic signals. Professions such as optometry (OPT) and physician assistant (PA), which also require substantial human capital investment, demonstrate tighter alignment between training requirements, reimbursement authority, and scalable care models. That alignment is reflected in more predictable earnings growth, clearer career progression, and sustained workforce expansion. Audiology's comparatively weaker alignment across these same mechanisms helps explain its distinctive workforce and economic patterns without implying differences in clinical value or societal importance.

By applying this framework, the sections that follow examine not whether audiology should function as an autonomous profession, but whether current economic structures allow it to do so in practice. The goal is not to prescribe reform, but to diagnose where misalignments exist between educational investment, institutional authority, and labor market outcomes—misalignments that shape the profession's present status and long-term sustainability.

### Human Capital Investment

Human capital investment reflects the time, financial cost, and opportunity cost required to enter a profession. In health economics, extensive investment in education and training creates an implicit economic expectation that lifetime returns—through earnings growth, career flexibility, and professional authority—will scale with that investment. These expectations are not normative or aspirational; they are revealed through observed labor market behavior and institutional valuation over time.

When returns to human capital are aligned with training intensity, professions tend to demonstrate stable labor supply, sustained workforce entry, and long-term retention. When they are not, labor markets respond predictably. In audiology, this misalignment is observable: a 3-year (2022–2024) mean annual salary of approximately $92,477, set against doctoral debt loads commonly exceeding $120,000, produces a debt-to-income ratio that compares unfavorably with the 3-year (2022–2024) mean annual salary for OPT ($139,013) and PA ($130,887)—professions with comparable or lesser training intensity (see Appendix A, Bray and Amlani 2 ). Prospective entrants reassess career choice, geographic and practice mobility decline, incentives for advanced training or specialization weaken, and attrition increases across the professional lifecycle.

Within this framework, human capital investment functions as a baseline against which other economic signals are interpreted. Reimbursement authority, pricing power, productivity, and earnings growth derive their significance from whether they generate returns commensurate with the cost and duration of training. Persistent misalignment between investment and return therefore signals structural constraints on professional status—constraints that shape workforce behavior regardless of clinical competence or societal need.

### Pricing and Reimbursement Authority

The second dimension is pricing and reimbursement authority—the degree to which a profession can independently value, bill, and deploy its services across payers and care settings. Professions with broad reimbursement recognition can participate in multiple payment categories, adapt services to evolving care models, and diversify revenue streams. This authority is shaped not only by clinical competence but by how services are defined, categorized, and embedded within payer benefit structures.

Audiology and speech-language pathology (SLP) offer a useful comparison because they occupy adjacent clinical domains and require graduate-level training, yet differ meaningfully in how their services are institutionally recognized and reimbursed. Although neither profession has practitioner status under Medicare, their services are treated differently within Medicare benefit structures. SLP services are embedded within long-standing therapy benefit categories and are reimbursed across a wider range of diagnoses, care pathways, and settings, even as revenue streams remain subject to fluctuation due to varied payment models and ongoing legislative changes.

Audiology's reimbursement pathways, by contrast, remain more narrowly defined and historically concentrated in diagnostic testing, with much of audiologic care delivered through nonreimbursed, technology-linked service models. While the Center for Medicare and Medicaid Services (CMS) established a limited direct-access exception in 2023—permitting patients to access certain diagnostic tests for nonacute hearing conditions without a physician order, once per 12-month period—this modification does not extend to rehabilitative services and therapeutic care, nor does it confer practitioner status under Medicare. 3 The structural ceiling, therefore, remains: revenue generation is concentrated in a restricted band of diagnostic codes, and the broader scope of contemporary audiologic practice—chronic disease management, rehabilitation, and longitudinal care—falls largely outside covered categories. These structural features limit pricing authority and constrain revenue diversification, even as audiologists' clinical scope and training support broader roles in care delivery.

Moreover, professions whose services are narrowly defined or inconsistently reimbursed face structural constraints on revenue growth, scope expansion, and integration into care pathways—regardless of training intensity or clinical sophistication. Within this framework, pricing and reimbursement authority serves as a central determinant of whether professional expertise can be translated into sustainable economic and workforce outcomes.

### Market Power and Substitutability

The third dimension is market power and substitutability. Market power reflects a profession's ability to influence how its services are priced, accessed, and deployed, as well as the degree to which its labor remains differentiated within healthcare markets. Professions with greater market power can command prices that reflect training intensity, clinical judgment, and accountability. Professions with limited market power face constrained pricing authority and weaker bargaining leverage, even when clinical expertise and responsibility are substantial.

Market power is shaped by broader healthcare market structures, including the extent of vertical integration across service delivery, financing, and distribution. In more integrated systems, professional services may be bundled, standardized, or mediated through organizational entities that control access, pricing, or volume. As a result, demand for healthcare services does not always translate directly into demand for autonomous professional labor; instead, value may be captured upstream, weakening the connection between professional judgment and economic valuation.

Substitutability is a primary mechanism through which market power is eroded. Services that can be readily replaced by alternative providers, technologies, or consumer-facing products tend to be priced more competitively and exhibit weaker professional leverage, regardless of underlying clinical value. By contrast, when services are clearly differentiated as professional labor—requiring specialized judgment, accountability, and scope-based authority—substitutability is reduced, supporting stronger pricing power and institutional standing.

Within this framework, market power and substitutability provide a lens for assessing whether a profession's services function as differentiated clinical labor or as interchangeable inputs within broader delivery systems. Persistent erosion of differentiation—through substitution, bundling, or externally anchored pricing—signals constrained professional authority and limits the ability of professional labor to translate training and expertise into sustainable economic and workforce outcomes.

### Productivity, Leverage, and Scalability

Productivity, leverage, and scalability together form the fourth dimension of professional status. In economic terms, productivity refers to the amount of valued clinical output generated per unit of professional labor. In healthcare, professional status is closely tied to whether productivity can increase beyond one-to-one service delivery. Professions that achieve higher economic standing typically develop care models that allow productivity gains through delegation, supervision, and team-based structures, enabling revenue growth without proportional increases in individual clinician labor.

Leverage describes the extent to which professional judgment can be extended across multiple patients, providers, or care processes—such as through supervision, protocol-driven care, or tiered service models. Scalability reflects whether delivery structures allow care capacity to expand while preserving quality, accountability, and professional authority. Professions constrained to linear, clinician-centric models face inherent ceilings on productivity, earnings growth, and workforce expansion, regardless of population demand.

In audiology, the predominant care model remains clinician-centric and encounter-based. The diagnostic evaluation, hearing aid fitting, counseling, and follow-up management that constitute core audiologic practice are typically delivered through sequential, one-to-one interactions that are difficult to delegate without direct audiologist involvement. Unlike professions that have developed tiered models—in which doctoral-level clinicians supervise support personnel, interpret results across a patient panel, or provide consultative oversight of protocol-driven care—audiology's prevailing reimbursement and delivery structures have not incentivized comparable delegation. The result is that increases in patient volume translate primarily into increased individual clinician workload rather than into system-level productivity gains, constraining both revenue growth and the capacity to expand workforce entry points.

Emerging delivery innovations—including teleaudiology, audiology assistants, and community-based hearing screening programs—represent nascent pathways toward greater leverage and scalability. However, their capacity to alter the profession's economic structure remains limited by two persistent constraints. First, reimbursement frameworks have not yet consistently recognized supervisory or delegated audiologic care as independently billable, meaning that productivity gains from these models are not reliably captured as revenue. Second, the scope and deployment of audiology assistants remain inconsistently regulated across states, limiting the profession's ability to build standardized, scalable care teams comparable to those that support physical therapy or physician-led practices. Until these structural conditions change, emerging models function as operational adaptations within a constrained economic framework rather than as drivers of scalable professional growth.

Payment systems and care delivery structures play a central role in shaping these dynamics. When reimbursement models recognize and support supervisory, team-based, or tiered care, professions are better positioned to translate training and expertise into scalable productivity. In audiology, the absence of such recognition is particularly consequential: Medicare and most private payers do not reimburse for audiology assistant services, do not provide billing pathways for supervisory time, and do not distinguish between doctoral-level clinical judgment and technician-level task completion in their fee structures. When payment systems fail to differentiate by complexity, accountability, or supervisory function, the economic incentive to build leverage into care models is suppressed—and productivity remains tightly coupled to individual clinician time regardless of available workforce infrastructure.

Within this framework, productivity, leverage, and scalability indicate whether a profession's economic role is structured for sustainable growth or constrained by institutional arrangements that inhibit the extension of professional labor. Where these constraints persist, professional authority remains operationally limited, and workforce expansion becomes increasingly difficult to sustain. For audiology, resolving these constraints requires not only changes in clinical practice culture but structural shifts in how payers define, recognize, and reimburse team-based and delegated audiologic care—conditions that Audiology 2050 implicitly depends upon but which current economic signals suggest remain unrealized.

### Earnings and Wage Growth

Earnings and wage growth function as composite signals that integrate all prior dimensions of professional status. In health economics, compensation patterns are interpreted not as isolated individual outcomes, but as aggregated expressions of how healthcare systems and labor markets value professional labor. Wage levels, growth trajectories, dispersion across experience and specialization, and responsiveness to expanded scope collectively indicate whether a profession's economic authority aligns with its training, responsibilities, and role within care delivery.

When human capital investment, reimbursement authority, market power, and scalable productivity are aligned, earnings tend to exhibit predictable characteristics: sustained real growth over time, differentiation by experience and specialization, and lifetime income trajectories that reflect increasing professional authority. These patterns signal that professional judgment is independently valued, that services are reimbursed as labor rather than commodities, and that productivity gains are supported by prevailing delivery and payment structures.

When one or more of these dimensions are constrained, earnings become an early and reliable indicator of structural misalignment. Compressed wage distributions, modest growth over the clinical lifespan, and weak returns to specialization or experience suggest that professional labor is not fully valued or extensible within existing systems. In this sense, earnings serve as an indicator of professional status, reflecting whether autonomy, scope, and productivity are being operationalized in practice rather than articulated in policy or vision statements.

Taken together, earnings and wage growth provide the clearest summary measure of professional alignment because they translate upstream structural conditions into observable labor market behavior. As such, they offer a practical lens through which claims of professional status can be evaluated against how the healthcare system actually rewards professional labor.

As a framework, these dimensions clarify why alignment matters. When aligned, predictable outcomes follow: stable workforce growth, durable career pathways, integration into multidisciplinary care, and resilience to technological disruption. When misaligned, workforce strain, earnings compression, role ambiguity, and stalled professional advancement emerge. This framework now provides the analytic lens through which the following sections interpret audiology's current professional position, helping to explain why forward-looking initiatives—such as Audiology 2050—emphasize structural alignment across education, reimbursement, and service delivery. Without such alignment, even well-trained professionals struggle to translate vision into durable economic outcomes.

## Health Economic Application of Professional Status

The framework developed above operationalizes professional status through observable economic signals rather than credentials or stated scope. The sections that follow apply this framework directly to audiology, drawing on evidence synthesized elsewhere in this special issue to examine how human capital investment, reimbursement authority, market power, productivity, and earnings interact in practice. By shifting from conceptual structure to applied analysis, this section interprets how audiology's current economic signals reveal its professional position within the U.S. healthcare system and how misalignment across these dimensions contributes to the workforce patterns and earnings trajectories observed today.

### Human Capital Investment and Economic Return

Audiology requires substantial human capital investment. Doctoral education, extended supervised clinical training, licensure, and ongoing professional development collectively mirror—and in some cases exceed—the preparation demands of other doctoral-level healthcare professions. In economic terms, such investment establishes an expectation that lifetime earnings, career flexibility, and professional authority will scale accordingly. When training intensity increases without commensurate economic return, labor market responses are not idiosyncratic, but systematic.

As reported in Bray and Amlani 2 (this issue) and summarized in Table 1 , 4 audiologists earnings have not aligned with these expectations. Compared with peer health professions requiring comparable—or, in some cases, lower—levels of formal training, audiologists experience compressed earnings distributions, modest wage growth over time, and limited upside across the clinical lifespan. Importantly, these patterns persist beyond early career stages and across practice settings, indicating that the issue is not transitional.

**Table 1:** Three-year mean salaries and student loan debt, with corresponding 3-year average growth rates, across four professions. Salary data were derived from Economics of Audiology Wages (this issue), and student loan debt data were derived from Economic Implications of Audiology Student Debt (this issue)

Profession (BLS SOC) 4	3-y mean annual salary	3-y mean annual salary growth	3-y mean student loan debt	3-y mean student loan growth
Audiologists (29–1181)	$92,477	$1,944	$127,117	$2,521
Optometrists (29–1041)	$139,013	$2,613	$221,307	$1,674
Physician assistants (29–1071)	$130,887	$3,877	$115,700	$2,131
Speech-language pathologists (29–1127)	$92,643	$2,126	$51,715	$1,159

From a health economics perspective, this pattern is consistent with a depressed return on human capital investment rather than deficiencies in educational quality, clinical competence, or professional commitment. When returns to training are constrained, incentives for workforce entry, geographic mobility, specialization, and long-term retention weaken. Over time, these responses interact with rising educational debt to shape workforce behavior, contributing to the sustainability challenges documented elsewhere in this issue.

The consistency of these outcomes across regions and employment contexts points to structural constraints on reimbursement authority, pricing power, and scalable productivity. These constraints suppress the economic return to audiology's substantial investment in training and limit the profession's capacity to translate doctoral-level preparation into durable economic and workforce outcomes—setting the stage for the dimensions examined in the sections that follow.

### Reimbursement Authority and Pricing Power

Reimbursement authority is a core determinant of professional status within healthcare labor markets. Professions recognized as independent providers by public and private payers can price services directly, participate in multiple payment categories, and adapt to evolving value-based care environments. This authority signals institutional recognition of professional judgment, clinical accountability, and scope. In contrast, professions that lack direct reimbursement authority remain structurally dependent on referral pathways and intermediary billing arrangements, constraining both economic autonomy and professional deployment.

Audiology's constrained reimbursement authority has direct implications for pricing power and revenue diversification. Despite doctoral-level training and responsibility for complex diagnostic, rehabilitative, and chronic-care services, audiologists operate within payment structures that recognize only a narrow subset of professional activities or require physician mediation for patient access and billing. As a result, revenue generation remains concentrated in restricted service categories and legacy delivery models rather than reflecting the full scope of contemporary audiologic practice.

From a health economics perspective, limited pricing power functions as a systemic constraint on earnings growth and workforce sustainability. When services cannot be independently valued or reimbursed in proportion to training and clinical responsibility, compensation remains compressed and less responsive to specialization, experience, or expanded scope. Over time, this weakens the labor market signal associated with professional autonomy, discourages advanced role development, and contributes to the depressed return on human capital investment documented elsewhere in this issue. These constraints are structural rather than situational, affecting audiologists across practice settings and reinforcing the broader misalignment between educational preparation and economic authority.

### Market Power and Substitutability

Applying the framework of market power and substitutability to audiology reveals a profession whose clinical labor remains economically vulnerable despite substantial training and scope. Audiology exhibits incomplete differentiation of professional labor as an autonomous healthcare service, limiting pricing authority and institutional leverage across much of the healthcare market.

Historically, audiology's market power has been shaped by the close coupling of professional services with hearing aid delivery. When revenue generation is anchored to products rather than to independently reimbursed clinical judgment, professional labor becomes more susceptible to substitution. In such models, technological innovation and alternative delivery channels compete directly with the product component of care, while the professional component remains undervalued or implicitly bundled. This structure constrains the profession's ability to command prices that reflect training intensity, diagnostic responsibility, and longitudinal care management.

The emergence of over-the-counter and direct-to-consumer hearing technologies did not create this vulnerability; rather, it exposed a preexisting lack of economic differentiation between professional labor and device-based value within prevailing delivery models. Where audiologic services are not independently priced, reimbursed, and recognized as cognitive and clinical labor, market forces predictably reallocate value toward products that can be obtained without professional mediation. From a health economics perspective, this reflects high substitutability of professional services as currently structured—not diminished clinical value or relevance. The result is suppressed pricing authority, weaker bargaining leverage, and constrained earnings growth.

Beyond technological substitution, audiology's market power has been further shaped by the evolving role of hearing aid manufacturers within downstream care delivery and payment structures. Over the past two decades, manufacturers have expanded beyond device production into vertically integrated ecosystems that include corporate retail channels, practice management platforms, financing mechanisms, and affiliations with third-party benefit programs. Within these systems, access to hearing care is often mediated upstream of the clinical encounter, influencing how services are bundled, framed, and priced.

From a labor economics standpoint, such vertical integration regulates downstream demand for professional labor. Price anchoring and bundled benefit designs limit audiologists' ability to independently price and differentiate services, even as population-level demand for hearing technology grows. Consequently, increases in device utilization do not reliably translate into proportional increases in demand for autonomous professional audiologic services.

The erosion of independent practice ownership provides the most direct empirical measure of this dynamic at the market level. Prior to 2004, approximately 49% of hearing aids supplied in the U.S. market were dispensed through independently owned practices; by 2023, that share had fallen below 15%, while manufacturer-owned retail outlets had grown to account for approximately 38% of hearing aid supply. 5 The number of independently owned audiology practices declined in parallel—from an estimated 5,060 in 2004 to approximately 2,000 by 2024—driven in part by direct manufacturer acquisition of practices and in part by generational shifts in entrepreneurial entry. 4 From a labor economics perspective, this contraction is not incidental. Independent practice ownership is the primary mechanism through which audiology's product-linked revenue model can be converted into scalable professional income—the structural analog to the ownership patterns that allow optometry and veterinary medicine to capture value across both clinical and retail components of care. As independent ownership declines and distribution authority concentrates in manufacturer-owned and vertically integrated channels, the profession's aggregate pricing authority weakens, its ability to independently set service value diminishes, and the market power that would otherwise support earnings differentiation and workforce sustainability erodes in direct proportion. The data are consistent with vertical integration functioning not merely as a competitive inconvenience but as a structural displacement of the ownership channel through which doctoral-level audiology is most fully capable of translating its training into independent economic authority.

These dynamics have direct implications for workforce sustainability. When demand for professional labor is externally constrained through bundled pricing, vertically integrated access pathways, or manufacturer-aligned benefit structures, earnings growth and entrepreneurial entry are predictably muted. Over time, this suppresses incentives for independent practice ownership, limits geographic diffusion of services, and reinforces reliance on salaried employment models characterized by compressed compensation bands.

Importantly, these outcomes do not imply adversarial intent on the part of manufacturers. Rather, they reflect misaligned incentives within a device-dominant supply chain operating alongside a service-intensive profession. Manufacturers are rationally optimizing distribution efficiency and market access; however, the resulting structures shift economic authority away from professional labor and toward integrated systems that control pricing, access, and volume. Within such systems, audiologists increasingly function as inputs rather than as independent producers of healthcare services.

The optometry profession offers an instructive contrast: optometrists successfully defended market position by building a reimbursable diagnostic identity independent of device dispensing, while opticians absorbed the dispensing volume. Audiology faces an analogous dynamic with hearing aid specialists (HAS), but without the reimbursement floor that made optometry's two-tier model viable. Recent private practice market data provide direct evidence of this dynamic. 5 Analysis of aggregated transactional data from more than 1,130 U.S. private practice clinics — spanning both audiologist- and hearing aid specialist–owned practices, as distinguished from manufacturer-owned retail channels — found that third-party administrator (TPA) hearing aid volume declined at a historically unprecedented rate in 2025, with approximately 70% of that decline attributable to demand-side forces outside clinics' control, including Medicare Advantage plan disruptions and benefit restructuring. 5 Within this aggregate decline, the data show a clear performance-tier divergence: the highest-performing practices in the dataset reduced TPA unit volume at more than twice the rate of the broader clinic population and derived a substantially smaller share of total units from TPA channels, indicating a deliberate hedge against constrained reimbursement rather than passive exposure to demand-side losses. 5 This pattern is consistent with, though not a direct measure of, a broader dynamic in which audiology practices — more heavily weighted toward diagnostic and rehabilitative service value — appear better positioned to exit TPA dependency than HAS-oriented practices, whose revenue model remains more closely tied to device transaction volume; confirming this distinction directly would require provider-type-segmented data not available in the current dataset. The aggregate effect, regardless of provider type, is that practices retaining heavy TPA dependence are ceding an increasing share of the referral infrastructure through which patients enter hearing care — not as a result of inferior clinical value, but as a rational response to reimbursement structures that make TPA participation economically untenable. This pattern illustrates the substitutability dynamic at scale: when professional services cannot be independently priced and valued, market pathways reallocate patient volume toward whichever channels remain willing to absorb reimbursement pressure — regardless of provider type.

Viewed through the framework of market power and substitutability, audiology's economic position is, therefore, characterized by constrained differentiation of professional labor. When clinical judgment is economically tethered to products or externally governed benefit structures, substitutability pressures intensify, pricing authority weakens, and opportunities for service-dominant, scalable care models remain limited. The net effect is a labor market in which workforce growth is constrained not by insufficient population need, but by restricted institutional demand for autonomous professional services.

### Productivity, Leverage, and Scale

Applied to audiology, the productivity and leverage framework reveals a profession whose care delivery structure has not evolved in proportion to its training requirements. The diagnostic evaluation, hearing aid fitting, counseling, and follow-up management that constitute core audiologic practice remain predominantly encounter-based and one-to-one—a model that tightly couples revenue generation to individual clinician time and imposes a structural ceiling on both earnings growth and workforce expansion capacity.

The payment architecture is the primary constraint. Medicare and most private payers do not reimburse for audiology assistant services, do not provide billing pathways for supervisory time, and do not differentiate between doctoral-level clinical judgment and technician-level task completion in their fee structures. This absence of recognition suppresses the economic incentive to build leverage into care models—and ensures that productivity remains tightly coupled to individual clinician availability regardless of workforce infrastructure. Emerging delivery innovations such as teleaudiology, audiology assistants, and community-based screening programs represent nascent leverage pathways, but their ability to alter the profession's economic structure is constrained precisely because the productivity gains they generate cannot be reliably captured as billable revenue under existing payment rules. Until reimbursement recognizes supervisory and delegated care, these models remain operational adaptations rather than economic drivers.

The cumulative effect is compounding. Constrained productivity interacts with the reimbursement dependency and substitutability pressures documented in the preceding sections to reinforce workforce stagnation: when clinical volume cannot be leveraged through delegation, and when revenue pathways are externally constrained, the profession's capacity to absorb rising population demand is structurally bounded regardless of how many audiologists are trained. These constraints are not incidental—they are the specific conditions under which earnings emerge as a composite signal of professional status, integrating the cumulative effects of reimbursement authority, market power, and productivity limitations across the clinical lifespan.

### Earnings as a Composite Signal of Professional Status

In health economics, compensation patterns are interpreted not simply as individual outcomes, but as aggregated expressions of how healthcare systems and labor markets value professional labor. Wage levels, growth trajectories, dispersion across experience and specialization, and responsiveness to expanded scope collectively reveal whether a profession's economic authority aligns with its training, clinical responsibilities, and role within care delivery.

Evidence synthesized in this special issue, and consistent with national workforce surveys, indicates that audiology earnings exhibit a pattern characteristic of constrained professional authority. Three-year (2022–2024) mean compensation for audiologists—estimated at $92,477 annually—has grown modestly relative to inflation-adjusted expectations for doctoral-level health professions, and the distribution across experience and specialization remains notably compressed ( Table 1 ). In OPT and PA practice, compensation bands widen meaningfully with years of experience, specialization, and practice ownership—reflecting return on advanced clinical investment. In audiology, these differentials are substantially narrower, suggesting that the labor market does not reward expanded scope or accumulated expertise in proportion to training intensity. Notably, audiology's student loan debt grows by more dollars per year than salary does ($2,521 vs. $1,944, from Table 1 ) — meaning that even as the debt-to-income ratio remains roughly stable, the absolute gap between debt and income widens each year, sustaining a persistently high repayment burden rather than narrowing it. Conversely, SLP's debt-to-income ratio is favorable, with wages nearly double its student loan debt. Importantly, these characteristics persist across practice settings and employment contexts, indicating that they reflect systemic features of the labor market rather than isolated organizational practices or individual career decisions.

From a health economics perspective, this pattern represents a form of revealed valuation by the healthcare system. Regardless of stated commitments to access, quality, or population health, observed reimbursement structures, pricing constraints, and deployment models position audiology below professions with comparable training but greater economic authority. This positioning is not accidental—it is the predictable outcome of the structural conditions examined in the preceding sections: a narrow reimbursement footprint, high substitutability of service delivery, and limited mechanisms for productivity leverage. Earnings, thus, serve as an indicator of professional status—reflecting whether autonomy, scope, and productivity are being operationalized in practice rather than articulated in policy, statute, or vision statements. When this pattern is consistent across time, geography, and practice setting, the evidence is more compatible with structural than situational explanations.

Crucially, this interpretation does not imply diminished clinical value or societal relevance. Instead, it reflects constrained institutional power. When professional judgment cannot be independently reimbursed, when services remain vulnerable to substitution, and when productivity is tightly coupled to individual clinician labor, earnings will fail to scale with training intensity and responsibility. The resulting compensation profile is therefore not anomalous, but a predictable outcome of structural misalignment—one that helps explain the workforce constraints and sustainability challenges documented throughout this issue. In this sense, audiology's earnings profile does not merely reflect its current professional position; it actively reproduces it by constraining the workforce incentives, entry patterns, and retention dynamics that would otherwise support professional expansion.

## Relationship to Audiology 2050

Audiology 2050 articulates a future state in which audiologists function as autonomous healthcare providers fully integrated into comprehensive, value-based care pathways. From a health economics perspective, this vision can be understood as a testable proposition: if professional autonomy expands, reimbursement authority broadens, and service delivery shifts toward independently valued clinical labor, then observable economic signals—particularly earnings growth, wage dispersion, and workforce stability—should respond accordingly. The framework presented in this manuscript provides the analytic tools to evaluate that proposition not through aspirational language, but through the same economic signals that reveal professional status in practice.

The analysis presented here suggests that audiology has not yet crossed the economic thresholds required for the Audiology 2050 model to function. The evidence is consistent across dimensions: a debt-to-income ratio that is widening rather than stabilizing, a reimbursement footprint concentrated in diagnostic codes while rehabilitative and chronic-care services remain largely uncovered, professional labor that remains vulnerable to substitution through device-linked delivery models and vertically integrated benefit structures, and productivity constrained to clinician-centric encounters without viable pathways for leverage or scale. Taken together, these signals do not describe a profession on the cusp of autonomous integration—they describe one whose structural position must change materially before Audiology 2050's operating assumptions can be realized in practice.

Importantly, this assessment does not challenge the goals or intent of Audiology 2050. Rather, it identifies the specific economic conditions that must shift for the vision to move from aspiration to market reality. Professions that have achieved sustained earnings growth and workforce stability have done so when changes across taxonomy, revenue recognition, and labor deployment occurred in coordination—not in isolation. Nurse practitioners and physician assistants gained independent prescribing authority and expanded reimbursement recognition simultaneously; physical therapists achieved direct access legislation, doctoral-level entry requirements, and visit-based reimbursement structures in tandem. In each case, no single change was sufficient. Three structural thresholds are similarly consequential for audiology, and alignment across all three is required for durable change. First, federal practitioner recognition under Medicare—not simply diagnostic carve-outs, but reimbursement authority across the full scope of audiologic practice, including rehabilitative and chronic-care services—is a prerequisite for the revenue diversification and pricing authority that autonomous professional status requires. The regulatory foundation for this change is not absent; it is incomplete. Since 2007, audiologists have been required to hold individual National Provider Identification (NPI) numbers, and CMS policy already requires that audiologic services be billed under the audiologist's own NPI. The infrastructure of individual provider recognition exists; what remains is the reclassification that would convert it into full reimbursement authority. Second, a viable and consistently reimbursed service extender pathway is necessary to decouple productivity from individual clinician time, enabling the leverage and scalability that support earnings differentiation and workforce expansion. Developing tiered service models, expanding the utilization of audiology assistants in supervised care contexts, and establishing structured longitudinal care pathways for chronic hearing and vestibular conditions would increase scalability and productivity—while positioning the profession to capture the full economic value of reimbursement changes when they occur. Third, earnings trajectories must begin to reflect return on doctoral-level investment—with compensation bands that widen meaningfully with experience, specialization, and practice ownership, rather than remaining compressed across the clinical lifespan. Scope modernization and delivery model development must advance in conjunction with reimbursement recognition, not ahead of it; scope expansion without accompanying reimbursement pathways risks expanding clinical authority without improving economic viability, a pattern well documented in healthcare professions where scope changes have outpaced payer recognition. Progress on any single threshold will produce a limited effect; alignment across all three is required for the economic signals to shift in ways consistent with doctoral-level professional standing.

The consequences of this misalignment are already observable in private practice markets, 5 6 and are examined in detail in the companion synthesis of workforce expert perspectives in this issue (see Audiology in 2050). These market-level signals reinforce the conclusion that workforce supply constraints and reimbursement misalignment are not future risks—they are present-state conditions actively shaping how patients enter, and fail to enter, audiologic care. Each of the three structural thresholds identified above maps directly onto a mechanism driving this observed market behavior.

From a health economics standpoint, Audiology 2050 is best understood not as a description of present conditions, but as a specification of the structural alignment required for professional autonomy to be reflected in market behavior. The value of that specification is practical: it defines what progress looks like in measurable terms. For policymakers, it identifies the reimbursement reforms that would materially alter professional standing. For educators, it clarifies the debt-to-income dynamics that shape workforce entry and retention independent of curricular quality. For professional leaders, it establishes that incremental reforms—those that do not alter the underlying economic signals—will continue to yield limited results regardless of clinical or advocacy momentum. Progress toward Audiology 2050 should therefore be evaluated not only by changes in statute, scope, or institutional recognition, but by whether the economic signals examined in this manuscript begin to shift in ways consistent with doctoral-level professional standing. Until they do, the gap between audiology's aspirational positioning and its observed market behavior will persist—and the workforce challenges documented throughout this issue will remain structurally reproduced rather than resolved.

## Conclusion

Viewed through a health economics lens, audiology currently occupies an intermediate professional position within U.S. healthcare. It is neither a technical service occupation nor a fully autonomous doctoral profession in economic terms. Rather, it reflects a field characterized by substantial human capital investment and clinical sophistication, yet constrained by limited reimbursement authority, restricted pricing power, vulnerability to substitution, and limited opportunities for scalable productivity.

This positioning helps explain the persistent workforce challenges documented throughout this special issue, including constrained earnings growth, rising debt-to-income pressures, uneven workforce expansion, and difficulty sustaining advanced clinical roles at scale. Importantly, these outcomes are not anomalous; they are predictable consequences of misalignment between educational preparation, institutional authority, and market valuation. In this context, incremental or isolated reforms have yielded limited impact because they do not materially alter the economic signals that govern professional status. In health economics terms, audiology's current earnings profile effectively places a ceiling on realized autonomy, regardless of scope or training.

Clarifying audiology's present-state position is, therefore, essential—not to assign blame or diminish clinical value—but to establish a realistic baseline for evaluation. For policymakers, educators, and professional leaders, this clarity provides a necessary reference point against which future initiatives can be assessed. Progress toward autonomy should be measured not only by changes in statute, scope, or vision, but by whether observable economic signals—earnings trajectories, workforce stability, and service valuation—respond in ways consistent with doctoral-level professional standing.

From a health economics perspective, initiatives focused on federal provider recognition, state-level scope modernization, and greater professional alignment can be understood as efforts to correct the structural misalignments identified in this analysis—misalignments that currently suppress pricing authority, earnings growth, and workforce sustainability. Only when these economic signals begin to shift will audiology's professional status, as revealed by the healthcare system itself, move decisively from aspiration to realization.
